# Reframing faculty development for digital transformation and generative AI in higher education

**DOI:** 10.3389/fmed.2026.1864520

**Published:** 2026-07-03

**Authors:** Zhitao Hou, Jing Chen, Hao Zhang

**Affiliations:** College of Basic Medical and Sciences, Heilongjiang University of Chinese Medicine, Harbin, Heilongjiang, China

**Keywords:** digital competence, educational digitalization, faculty development in higher education, faculty development model, generative artificial intelligence

## Abstract

As educational digitalization continues to deepen and generative artificial intelligence rapidly enters higher education teaching contexts, faculty professional development is undergoing a profound transformation from tool use to pedagogical redesign and from individual adaptation to organizational change. However, current faculty development practices still tend to substitute standardized training for differentiated support, making it difficult to address the diverse needs of teachers at different career stages in digital competence, curricular innovation, disciplinary integration, and academic leadership. To enhance the transparency of the framework-building process, this study was informed by explicit review questions, database-based literature retrieval, and thematic synthesis. Building on scholarship on teacher digital competence, teacher professional development theory, and the practice of digital transformation in higher education, with Heilongjiang University of Chinese Medicine serving as the institutional context for framework formulation, this paper offers a systematic account of the stage-specific developmental tasks of university faculty in the digital era and advances a multi-level, developmental framework. The framework is organizationally supported by coordination across three institutional levels—university, school, and department—structured around four developmental stages: entry and adaptation, stable development, deep integration, and academic leadership, and implemented through five pathways: blended professional learning, cross-boundary linkage, community-based mutual learning, diagnostic mentoring, and evidence-informed practice. The study contends that faculty development should move beyond a narrow focus on digital tool training and instead emphasize the systematic development of capacities related to pedagogical improvement, disciplinary reconfiguration, digital ethics, and organizational leadership. This framework helps enhance the stage-specific relevance, practical explanatory power, and organizational operability of faculty development, while also providing a theoretical reference for designing faculty development systems in digitally transformed universities.

## 1 Introduction

Global higher education is shifting from technology-assisted instruction to the digital reconfiguration of teaching. Digital technologies are no longer merely auxiliary tools for content delivery, but are increasingly embedded throughout the entire process of curriculum design, learning analytics, teaching assessment, resource production, and faculty collaboration. The European Union’s DigCompEdu framework emphasizes that teachers’ digital competence should encompass multiple dimensions, including professional engagement, digital resources, teaching and learning, assessment and feedback, empowering learners, and facilitating learners’ digital competence (1); UNESCO’s ICT Competency Framework for Teachers further argues that the enhancement of teachers’ technological capacity should proceed in coordination with curriculum, pedagogy, organizational governance, and continuous professional learning (2); the OECD likewise regards standards for teachers’ digital competence, incentives for continuing professional development, and institutional ecosystem support as key levers for digital transformation in education (3). These perspectives indicate that international higher education research has increasingly come to understand teachers’ digital development as an organizational, sustained, and contextualized form of professional growth rather than a one-time technical training event. However, existing faculty development practices in higher education still remain largely at the level of broad advocacy, with insufficient attention to the actual needs of teachers at different developmental stages and an underdeveloped understanding of how digital literacy, pedagogical knowledge, and disciplinary knowledge should be integrated. Especially as generative artificial intelligence rapidly enters the classroom, teachers are required not only to address emerging issues such as curriculum redesign, assessment reform, academic integrity, and data ethics, but also to respond to the instructional and organizational pressures generated by changing student learning practices. Existing studies show that the focus of research on university teachers’ digital competence is shifting from whether they can use digital tools to whether they can develop stable, reflective, and transferable digital teaching practices within specific disciplinary and instructional contexts (4–7). Accordingly, this review was guided by three questions: (1) What stage-specific developmental needs of university faculty have been emphasized in the literature on digital transformation? (2) What institutional support structures and professional development pathways have been most consistently identified? (3) How can these findings be synthesized into a transferable framework while remaining anchored in the institutional context of Heilongjiang University of Chinese Medicine? In response to these questions, the framework presented in this study was derived through a structured reading and synthesis of the relevant literature, so that the key analytical claims reported in the following sections remain identifiable through specific supporting sources. This paper proposes a “three-tier, four-stage, five-pathway” development framework, organized around collaboration across the university, school, and department as three institutional levels, structured by four progressive developmental stages—entry adaptation, stable development, integrative deepening, and academic leadership—and implemented through five pathways: blended professional learning, cross-boundary linkage, community-based mutual learning, diagnostic mentoring, and evidence-informed practice, thereby providing an operational theoretical reference for faculty digital professional development in higher education (4).

## 2 Review objectives, research questions, and methodological approach

This review addressed three objectives: to identify the stage-specific developmental needs of university faculty under digital transformation; to synthesize the institutional support structures and implementation pathways reported in the literature; and to integrate these findings into a framework formulated in the institutional context of Heilongjiang University of Chinese Medicine. To improve methodological transparency, the literature collection and organization process was structured with reference to the Population–Concept–Context (PCC) principle, and the reporting logic was aligned, where applicable, with major transparency items from PRISMA-ScR.

### 2.1 Data sources, search strategy, and eligibility

Relevant literature was retrieved from four major databases widely used in education and interdisciplinary higher education research: Web of Science Core Collection, Scopus, ERIC, and PubMed. Search terms were organized around four clusters: faculty or teacher development (e.g., faculty development, teacher professional development), digital competence (e.g., digital competence, digital literacy), higher education context (e.g., higher education, university teachers), and technological transformation (e.g., digital transformation, blended learning, generative AI, artificial intelligence). Backward reference checking of key studies was also undertaken to improve coverage. Studies were included when they addressed higher education teachers or faculty members, focused on digital competence or faculty development, and discussed pedagogical, organizational, or ethical implications relevant to digital transformation. Studies focusing exclusively on K–12 settings, student-only outcomes, or purely technical descriptions without implications for faculty development were excluded from the interpretive synthesis. This selection logic was used to ensure that the literature base directly informed the analytical dimensions subsequently used to construct the framework.

### 2.2 Study selection, synthesis, and framework construction

The collected studies were read iteratively and analyzed through thematic synthesis. Recurring concepts were coded under three integrative dimensions: developmental needs across faculty career stages, institutional levels of organizational support, and professional development pathways. These codes were then compared and consolidated to generate the final framework. The “three-tier” component was derived from the recurrent organizational distinction between university-, school/college-, and department-level support; the “four-stage” component emerged from patterns in career-stage-specific developmental tasks; and the “five-pathway” component was synthesized from the principal implementation strategies repeatedly described in the literature. This procedure clarified the literature collection and analytical process and provided structured support for the framework presented in this manuscript.

## 3 Stage-specific developmental needs of faculty professional development in the context of digital transformation

### 3.1 Entry and adaptation: from technological familiarity to pedagogical integration

Early-career university teachers typically demonstrate a relatively high level of acceptance of technology, yet their challenge often lies not in whether they are willing to use digital tools, but in how to effectively integrate technology, pedagogy, and disciplinary content. Masoumi and Noroozi (8) reported that early-career teachers frequently face challenges in digital teaching, including restricted resource availability, inadequate institutional support, insufficient technical and pedagogical guidance, and heavy workloads, whereas mentorship and professional identity formation constitute crucial conditions for the development of their digital competence. In other words, what junior faculty need most is not isolated “software instruction,” but integrated support that embeds technology, pedagogy, and disciplinary content within authentic course contexts. This implies that higher education institutions should shift from “induction training” to “instructional entry support” in the development of newly appointed faculty. The core of new faculty development should not merely be to help them adapt to procedural aspects of the job, but rather to help them build capacities for course design, classroom interaction, the identification of learning evidence, and reflective teaching in digital environments. DigCompEdu regards teaching, assessment, and learner empowerment as core components of digital competence (1), suggesting that effective technology integration should be understood as a pedagogical and disciplinary process rather than as mastery of isolated tools. Accordingly, faculty development at the entry and adaptation stage should focus on three areas: first, building foundational digital teaching capacities, including the use of course platforms, the organization of digital resources, basic learning analytics, and the responsible use of generative AI; second, embedding digital tools directly into course design, classroom questioning, formative assessment, and feedback revision through microteaching, workshops, and demonstration-based observation; third, reducing the cost of trial and error in digital teaching for new faculty through mentoring systems and peer support. Studies indicate that technological support, when disconnected from actual course practice, readily leads to fragmented learning; by contrast, embedded, contextualized, and feedback-driven forms of development are more effective in helping new faculty move from operational competence to pedagogical competence (9, 10). Taken together, these studies provide the main evidentiary basis for identifying pedagogical integration, mentoring, and contextualized support as defining needs of the entry and adaptation stage.

### 3.2 Stable development: from tool use to curricular innovation

At the stage of stable development, faculty are usually capable of handling teaching tasks independently, but their growth is often constrained by limited instructional innovation, weak capacity for digital course redesign, and insufficient efficiency in the coordination of teaching and research. Research suggests that gains in faculty digital competence do not necessarily lead to high-quality teaching practice; the crucial issue is whether teachers can create digitally supported instructional designs that are coherently organized around course goals, learning activities, and assessment strategies (5–7). Especially in a context where blended learning is becoming the norm, digital teaching no longer simply means moving the classroom online, but instead requires teachers to redesign learning processes, interaction mechanisms, and methods of evidence collection (9). From this perspective, the key developmental task for faculty at the stable development stage is to move from being users of digital tools to becoming designers of digital courses. The HeDiCom framework emphasizes that the digital competence of higher education teachers encompasses not only teaching practice itself, but also dimensions such as empowering students to participate in the digital society, teachers’ own digital literacy, and continuous professional learning (4). This suggests that the mid-career development of higher education faculty should not focus solely on training in classroom software, but should instead incorporate course redesign, the optimization of learning experiences, the cultivation of students’ digital competence, and teachers’ own capacity for continuous learning within a unified framework. For faculty at this stage, development efforts should focus primarily on three categories of competence. First, course design competence, that is, the capacity to reconstruct blended instructional processes in light of student characteristics and course goals, and to arrange preparation, interaction, discussion, collaboration, and assessment tasks appropriately. Second, evidence-driven improvement competence, namely the ability to adjust teaching on the basis of learning analytics, classroom data, and student feedback (11, 12). Third, research translation competence, namely the ability to connect curriculum reform with teaching research and to transform practical problems into topics for educational reform, course-based publications, or project outcomes. Studies have shown that blended professional development programs in higher education can positively strengthen teachers’ reflective practice, awareness of instructional design, and confidence in digital teaching, provided that such training is continuously tied to actual course practice rather than delivered through one-time intensive instruction (9, 13). These findings support the interpretation of curricular innovation, evidence-informed improvement, and research translation as core developmental priorities at the stable development stage.

### 3.3 Integrative deepening: from digital competence to disciplinary reconfiguration

Once teachers enter the stage of integrative deepening, their focus is no longer on the implementation of individual courses, but on how digital technology can genuinely transform the pedagogical logic of the discipline, the representation of knowledge, and the organizational structure of learning. The HeDiCom framework explicitly states that the mature form of faculty digital competence is manifested not merely in individual technical proficiency, but in the ability to embed digital practices into teaching innovation, student empowerment, and systems of professional learning for faculty (4). In other words, the central issue for teachers at this stage is to achieve deep coupling between technology and the discipline, rather than merely layering technology onto the outer surface of existing classrooms. There are three principal challenges at this stage. First, although some teachers possess extensive teaching experience, entrenched instructional habits make them more likely to regard digitalization as an auxiliary enhancement rather than as a restructuring of instructional architecture. Second, disciplinary specificity means that technology integration cannot follow a uniform template, and different fields vary substantially in their reliance on simulation, data visualization, collaborative platforms, virtual laboratories, or generative AI (5–7). Third, the deepening of technology use brings new ethical and governance issues, including the boundaries of data use, algorithmic bias, fairness in assessment, and the protection of student agency. In constructing its framework, HeDiCom has already incorporated ethical competence in digital society as a key dimension, indicating that digitally mature teachers must not only be capable of innovation, but also be able to discern technological risks (4). Therefore, faculty development at the stage of integrative deepening should be upgraded from “training courses” to “innovation platforms.” Higher education institutions can encourage faculty to advance technology integration in parallel with curriculum reform, textbook updating, and assessment transformation through smart courses, virtual teaching-research activities, interdisciplinary projects, and AI-assisted teaching experiments. Rather than repeatedly offering generic software training, it would be more effective to establish problem-centered, project-based professional learning mechanisms that organize interdisciplinary collaboration around specific questions such as how to redesign case-based teaching with generative AI, how to introduce virtual simulation into laboratory courses, or how to use learning analytics to optimize formative assessment. Only in this way can teachers truly develop digital teaching innovation capacities that are disciplinary, transferable, and demonstrable (4, 14).

### 3.4 Academic leadership: from individual excellence to organizational change

Faculty who enter the stage of academic leadership have often already developed strong influence in teaching, research, or disciplinary development, and their digital development tasks accordingly change: the priority is no longer to compensate for individual skill gaps, but to transform personal experience into organizational capacity, institutional resources, and demonstrative influence. The OECD particularly stresses that standards, incentives, and support ecosystems must be developed simultaneously in relation to teachers’ digital competence (3); this means that genuinely advanced teacher digital development cannot depend on individual effort alone, but also requires teachers to exercise leadership in course communities, school governance, resource sharing, and quality enhancement mechanisms. At this stage, the focus of faculty development should shift from “individual advancement” to “organizational mobilization.” On the one hand, high-level faculty should serve as designers and catalysts of institutional digital teaching reform, for instance by leading the development of exemplary courses, promoting the digital reconfiguration of disciplinary course clusters, and mentoring junior faculty in evidence-informed pedagogical research. On the other hand, they must also possess the capacity to evaluate emerging technologies prudently, especially in the context of the rapid diffusion of generative AI, where faculty leaders should attend to both instructional innovation and ethical norms so as to avoid allowing a logic of efficiency to override educational values. Current research suggests that centers for teaching and learning in higher education are under increasing pressure to maintain balance in the AI era, as they must both encourage faculty to experiment with new tools and guard against risks including opacity, injustice, and dehumanization (15). It follows that faculty development at the stage of academic leadership should focus on four capacities: first, platform-building capacity, namely the ability to transform high-quality personal experience into course resources, case repositories, and organizational mechanisms; second, cross-boundary collaborative capacity, namely the ability to connect schools, disciplines, technical departments, and external collaborative networks; third, evidence-informed decision-making capacity, namely the ability to support teaching governance through the use of course data, instructional evidence, and student feedback (11); and fourth, normative leadership capacity, namely the ability to develop transferable institutional experience in AI use, data ethics, and academic integrity (3, 16, 17). Only when highly accomplished faculty are able to promote organizational learning rather than remaining at the level of individual achievement can digital transformation move from localized innovation to systemic improvement. To improve the traceability of the analytical claims presented above, Table 1 summarizes the stage-specific developmental needs, support strategies, and representative references supporting each category of the proposed framework.

**Table 1:** Stage-specific developmental needs and support strategies for faculty professional growth under digital transformation.

Developmental stage	Core focus	Key challenges	Development priorities	Support strategies	Expected outcome	References
Entry and adaptation	Pedagogical integration	1. Fragmented support; 2. Heavy workload; 3. Weak teaching–technology connection;	1. Foundational digital teaching; 2. Course design; 3. Reflection	1. Mentoring; 2. Peer support; 3. Microteaching; 4. Workshops	Operational competence → Pedagogical competence	(1, 8–11)
Stable development	Curricular innovation	1. Limited innovation; 2. Weak redesign; 3. Low teaching–research coordination	1. Course design; 2. Evidence-driven improvement; 3. Research translation	1. Blended PD; 2. Learning analytics; 3. Student feedback; 4. Course-based practice	Digital tool user → Course designer	(4–7, 10, 12, 13, 18)
Integrative deepening	Disciplinary reconfiguration	1. Entrenched habits; 2. Disciplinary variation; 3. Ethical risks;	1. Disciplinary integration; 2. AI-enabled redesign; 3. Ethical discernment;	1. Innovation platforms; 2. Interdisciplinary collaboration; 3. Smart courses; 4. AI-assisted experiments	Transferable disciplinary innovation	(4–7, 14)
Academic leadership	Organizational change	1. Mechanism building; 2. Balancing innovation and ethics; 3. Governance pressure	1. Platform building; 2. Cross-boundary collaboration; 3. Evidence-informed leadership; 4. Normative leadership	1. Exemplary courses; 2. Mentoring; 3. Governance participation; 4. Resource sharing	Organizational learning → Systemic improvement	(3, 12, 13, 16)

## 4 Reconstructing the “three-tier, four-stage, five-pathway” framework for faculty development in higher education

### 4.1 Three-tier coordination: replacing isolated training with organizational alignment

Faculty digital professional development is highly dependent on organizational conditions; relying solely on institution-wide standardized training often fails to address disciplinary and developmental differences, whereas relying entirely on spontaneous initiatives at the school or departmental level can easily result in fragmented resources and uneven quality. On this basis, this paper proposes a three-tier coordination mechanism involving the university, the school, and the department. At the university level, the main responsibilities include institutional design, the construction of resource platforms, the formulation of developmental standards, and quality evaluation; at the school level, the focus is on disciplinary translation, project organization, and the cultivation of core faculty; at the department or teaching-research unit level, the task is to embed faculty development into routine curriculum construction, peer feedback, and classroom improvement. The OECD identifies ecosystem support as an essential condition for the development of teachers’ digital competence (3), while HeDiCom similarly stresses that higher education institutions must develop organizational visions and support systems that correspond to faculty digital competence (4). The value of this three-tier structure lies in shifting faculty development from isolated activities to a sustained institutional mechanism. The university ensures the stability of the “supply side,” the school ensures the adaptability of the “translation side,” and the department ensures the continuity of the “practice side.” These three levels should not operate as a mere division of labor; instead, they should form a closed loop involving data sharing, resource feedback, and outcome evaluation. For example, the university may provide a unified digital teaching platform and training certification system, on the basis of which schools design discipline-specific workshops, while departments consolidate learning outcomes into everyday improvement practices through open classes, peer observation, and course review. This design better aligns with the developmental logic of faculty professional growth in higher education than isolated lectures or one-time interventions.

### 4.2 Four-stage progression: aligning developmental priorities with career stages

Compared with traditional classifications, this paper favors a four-stage nomenclature consisting of entry and adaptation, stable development, integrative deepening, and academic leadership. This naming scheme reduces the administrative overtones of evaluation and better emphasizes faculty development as a dynamic professional process rather than a fixed category. The four stages do not suggest that faculty development proceeds only in a linear fashion; instead, they are used to characterize the main developmental tasks and support priorities that different faculty members are likely to face in the context of digital transformation: early-stage faculty need entry support, mid-stage faculty need course innovation, advanced faculty need disciplinary integration, and leading faculty need organizational leadership. Relevant research has shown that the development of teachers’ digital competence is jointly shaped by career stage, institutional environment, resource support, and opportunities for professional learning; therefore, tiered support is more effective than uniform training in improving the efficiency of faculty development (4). The four-stage progression also suggests that modes of evaluation should vary accordingly. At the entry adaptation stage, evaluation may focus on course design, classroom implementation, and reflective improvement; at the stable development stage, attention may be directed to course redesign, student engagement, and pedagogical research outputs; at the integrative deepening stage, the focus may be on cross-technology integration, shared resource development, and demonstrative dissemination; and at the academic leadership stage, emphasis should be placed on team leadership, mechanism construction, and policy influence. If evaluation standards fail to differentiate among developmental stages, all faculty may end up being measured against the same yardstick, which ultimately undermines the relevance of training and faculty motivation to engage.

### 4.3 Five pathways in concert: supporting sustained growth through multiple routes

**Figure 1:**
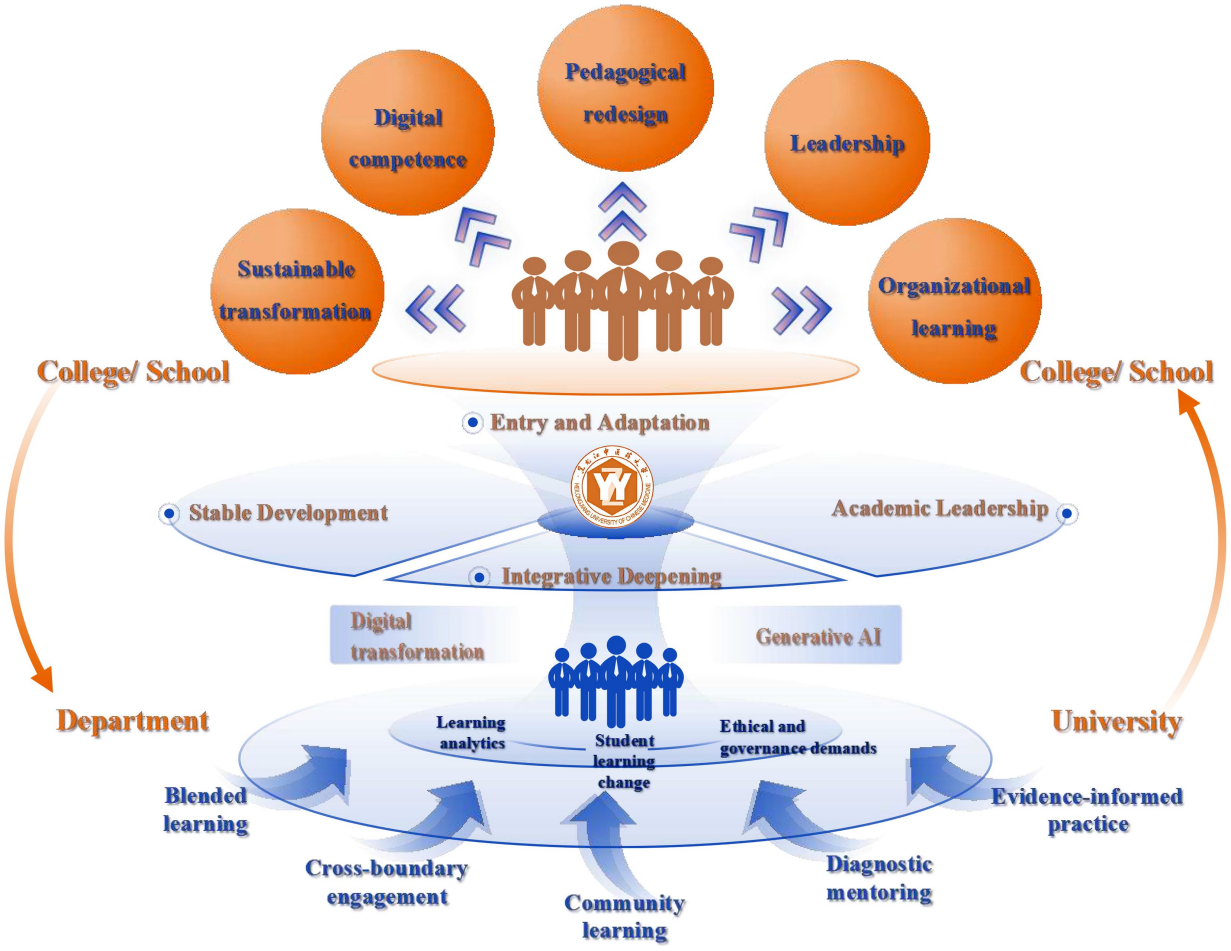
Conceptual framework of faculty development for digital transformation and generative artificial intelligence in higher education. The figure presents a faculty development framework developed in the institutional context of Heilongjiang University of Chinese Medicine and informed by its experience in teacher development and training. The framework integrates three levels of institutional support—university, school/college, and department—with four progressive stages of faculty development: entry and adaptation, stable development, integrative deepening, and academic leadership. It also identifies five implementation pathways: blended professional learning, cross-boundary linkage, community-based mutual learning, diagnostic mentoring, and evidence-informed practice. Together, these elements illustrate how faculty development can move from digital tool training toward pedagogical redesign, disciplinary integration, digital ethics, and organizational leadership.

At the level of implementation, this paper reconceptualizes “online + offline,” “inside campus + outside campus,” “collective + self-directed,” “training + guidance,” and “theory + practice” as five pathways more suitable for academic articulation, namely blended professional learning, cross-boundary linkage, community-based mutual learning, diagnostic mentoring, and evidence-informed practice. First, blended professional learning highlights the combination of online flexibility and in-person depth of interaction. Studies on blended professional development for teachers suggest that this model better enables teachers to connect external learning with local practice and to sustain engagement over time (9, 18). Second, cross-boundary linkage emphasizes connecting on-campus training with external academic networks, industry platforms, and inter-institutional collaboration. Digital teaching innovation in higher education rarely arises spontaneously within closed institutional settings; instead, it is continually stimulated through inter-organizational exchange, comparative case analysis, and interdisciplinary collaboration. Third, community-based mutual learning focuses on fostering the sharing of experience through teaching communities, virtual teaching and research groups, and communities of practice. Relevant literature indicates that community-based models can reduce teachers’ sense of isolation in digital teaching, promote knowledge sharing and continuous improvement, and are particularly suitable for faculty development in online, blended, and digital learning environments. Fourth, diagnostic mentoring emphasizes combining concentrated training with personalized support. Standardized instruction alone is unlikely to reach the practical problems teachers face in specific courses, whereas mentoring, peer feedback, instructional coaching, and project-based guidance are better positioned to meet differentiated needs (14). Fifth, evidence-informed practice emphasizes linking theoretical learning, classroom experimentation, data feedback, and reflective revision into a closed loop. Studies on learning analytics indicate that teaching data are not limited to student management purposes, but can also provide evidence to help teachers improve classroom interaction, assessment design, and feedback quality (11). These five pathways are not intended to replace one another, but rather to jointly constitute a support network for faculty digital professional growth (see Figure 1).

## 5 Supporting mechanisms for framework implementation

### 5.1 Enhancing developmental precision through diagnostic assessment

One major reason for the limited effectiveness of faculty development is that training provision often comes before a proper diagnosis of needs. When implementing the “three-tier, four-stage, five-pathway” framework, higher education institutions should first establish digital competence profiles for faculty in order to distinguish their respective strengths and weaknesses in areas such as technology use, course design, assessment reform, digital ethics, and organizational leadership. DigCompEdu and HeDiCom can both serve as valuable references for higher education institutions seeking to design contextually adapted diagnostic instruments (4). The former is suitable for constructing a relatively comprehensive set of dimensions for teachers’ digital competence, whereas the latter is more closely aligned with teaching practice, student empowerment, digital literacy, and professional learning in higher education contexts. Only by placing diagnosis before intervention can training become genuinely tiered and differentiated, rather than uniformly applied in form alone.

### 5.2 Replacing single time-based metrics with practice-based outcomes

In faculty development for digital transformation, simply counting the number of training hours is no longer sufficient to reflect the actual extent of improvement. More effective evaluation should attend to whether teachers have completed course redesign, developed high-quality digital resources, improved classroom interaction, optimized assessment on the basis of evidence, and contributed to team development. In other words, the focus of evaluation should shift from “what training has been attended” to “what teaching practice has been changed.” Studies on technology-enabled teacher professional development indicate that truly effective programs typically involve active learning, collaborative learning, contextual embeddedness, and sustained support, and that these features should ultimately be visible in teaching practice itself (17).

### 5.3 Building a sustainable development mechanism grounded in digital ethics

The growing diffusion of generative artificial intelligence has expanded the agenda of faculty development from competence enhancement to normative governance. In advancing faculty digital development, higher education institutions should not emphasize efficiency and innovation alone, but should simultaneously strengthen digital ethics, data responsibility, the identification of algorithmic bias, academic integrity, and the protection of student agency. HeDiCom has already included relevant ethical competences within its digital competence framework for higher education teachers (4), while UNESCO’s ICT Competency Framework for Teachers similarly stresses that technology use must be aligned with educational aims and institutional responsibilities (2). From this perspective, the hallmark of mature faculty development in the future will not be “how many AI tools are used,” but whether educators can maintain sound educational judgment among values, norms, and innovation.

## 6 Conclusion

Digital transformation changes not only the media of teaching, but also the underlying logic of faculty professional development in higher education. The existing materials have already perceptively recognized the differentiated needs faced by different groups of teachers in the process of digitalization, but if they are to be developed into an academic paper suitable for translation and publication in an English-language journal, further efforts are needed to remove slogan-like expressions and strengthen conceptual clarity, developmental logic, and literature support. On this basis, drawing on internationally recognized research on teachers’ digital competence, this paper proposes a “three-tier, four-stage, five-pathway” framework for faculty development and seeks to shift faculty development in higher education from standardized training to differentiated development, from technology transmission to pedagogical redesign, and from individual capacity building to the construction of organizational ecosystems. The future competitiveness of faculty development in higher education will lie not in the short-term adoption of the newest technologies, but in the capacity to build an iterative system of professional development that can help early-career faculty enter digital teaching, support established faculty in achieving curricular innovation, and enable core faculty to advance disciplinary integration and organizational leadership. In this sense, faculty development is not a subsidiary component of educational digitalization, but rather a decisive variable in determining whether digital transformation can truly reach the site of teaching, enter the structure of the curriculum, and improve student learning.
